# The narrative governance of life: Morality, melodrama, and the limits of biopower in western Indian efforts against sex selection

**DOI:** 10.1111/maq.12901

**Published:** 2024-11-29

**Authors:** Utpal N. Sandesara

**Affiliations:** ^1^ Division of General Internal Medicine and Health Services Research International Institute University of California Los Angeles Los Angeles California USA

**Keywords:** affect, biopower, morality, narrative, reproduction

## Abstract

Selective abortion of female fetuses is a widespread, illegal, and profoundly consequential form of family planning in contemporary India. In Gujarat state, public health campaigns against the practice rely on narratives exhibiting the hallmarks of melodrama: good–evil binaries, stock characters, emotional provocations, simplistic diagnoses, and inevitable triumphs. As biopolitical truths, such narratives resonate ethically and emotionally for people. By individualizing blame, obscuring structure, circumscribing discourse, and legitimizing authority, such narratives also exert many classic biopolitical effects. But they do not necessarily transform subjectivity or behavior, as biopower is often assumed to. Anti‐sex selection messaging illustrates how moralistic, sentimentalized interventions against potentially harmful practices can provoke strong responses without changing actions. In highlighting resonance as a relevant biopolitical limit, the not‐quite‐paradoxes of Gujarati public health narratives—encapsulation without accuracy, regulation without discipline, authority without efficacy, participation without transformation—suggest one approach for analyzing the governance of life without falling into determinism.

## Translated Abstract (Gujarati)

આજના ઇન્ડિયામાં ગેરકાયદેસર જાતિ પરીક્ષણ બાદ કરેલો સ્ત્રીભૃણનો ગર્ભપાત તે ખૂબ જ પ્રચલિત બની ચુક્યો છે. ગુજરાત રાજ્યમાં તે કૃત્યના વિરોધમાં ઊભાં થયેલાં જાહેર આરોગ્યનાં પગલાં “મેલોડ્રામા” શૈલીના 5 મૂળભૂત લક્ષણ ઉપર આધાર રાખે છે: સારા‐નરસાનું સરળ વિભાજન, રૂઢિગત પાત્રો, લાગણીઓની ઉશ્કેરણી, સરળ સામાજિક નિદાન, અને સારાપણાનો અનિવાર્ય વિજય. આવા લક્ષણવાળા સંદેશા સ્ત્રીભ્રુણના ગર્ભપાત માટે વ્યક્તિને દોષિત ઠરાવે છે, સામાજિક વ્યવસ્થાને છાવરે છે, સરકારની સત્તાને ટેકો આપે છે, અને જાહેર ચર્ચાને અમુક ક્ષેત્રમાં જ માર્યાદિત રાખે છે. આવા સંદેશા લોકોની ભાવના અને નીતિને સ્પર્શે પણ છે. પરંતુ તેથી આ પ્રકારના સંદેશા લોકોના વિચાર કે આચારમાં પરિવર્તન લાવશે જ, એવું જરૂરી નથી. સ્ત્રીભૃણના ગર્ભપાત વિરોધી ઝુંબેશો પુરવાર કરે છે કે હાનિકારક ગણાતી પ્રવૃત્તિઓ સામેના લાગણીવશ, નૈતિકવાદી પ્રયાસો લોકોમાં ખૂબ પ્રતિભાવ ઉપજાવે તેમ છતાં એવું બની શકે કે તેઓનું વલણ અને વર્તન બદલાય નહિ. વૈશ્વિક જાહેર આરોગ્યનો દાયકાઓથી ભણાવવામાં આવેલો સિદ્ધાંત ‐ કે માણસને માહિતી આપવાથી અભિગમ બદલાય, અને અભિગમ બદલાવાથી વર્તન બદલાય ‐ તે ક્યારેક આવી રીતે ખોટો પણ પડી શકે. સ્ત્રીભ્રુણના ગર્ભપાત વિરોધી ગુજરાતી ઝુંબેશોનો આ વિરોધાભાસ છે: સાચાપણા વિનાનો સારાંશ, પરિવર્તન વિનાનો હૃદયસ્પર્શ, બંધન વિનાનું નિયંત્રણ, અને સામર્થ્ય વિનાની સત્તા.

## INTRODUCTION

How do governance interventions against potentially harmful practices acquire ethical and emotional resonance? And how can such interventions fail to change behaviors despite resonating deeply?

In considering these questions, I keep returning to a summer evening in 2014. The setting was Mahesana, a district capital in western India's Gujarat state. Three hundred of us had crammed into a sweltering event hall. Around me sat a dozen‐odd women from my neighborhood. This “ladies’ event,” one chuckled, would “reveal the truth” about my research topic.

I was early in what would become 16 months of fieldwork on anti‐female prenatal sex selection.^1^ During the 2010s, roughly 400,000 sex‐selective abortions took place in India annually, with 8% of Gujarat's anticipated female births going “missing” (Kulkarni, 2020, 16). The 2011 census showed Gujarat had 0.890 girls per boy aged 0–6; Mahesana's figure was 0.760.^2^ Because prenatal sex determination was illegal, every missing girl reflected a family obtaining clandestine sex detection ultrasound and selective  abortion.

My neighbors knew the issue well. None was surprised by what unfolded once curtains opened on a one‐act play. A middle‐class couple sat eating breakfast. We howled when our neighbor Champa‐ben, playing the husband's mother, tottered onstage.^3^ A familiar plot took shape. The young woman was pregnant. The mother‐in‐law, desiring only a boy, insisted on sex determination.

We could readily infer the mother‐in‐law's motives. Gujarati families pursued sex selection for reasons broadly consistent from Beijing to the Balkans.^4^ Father–son connections structured descent, inheritance, and intergenerational relations. Men usually remained with parents after marriage, while women joined husbands’ families. Sons ostensibly guaranteed economic security and social recognition; daughters, financial and emotional liability. Given the commonplace perception that sonless elders faced material precarity and social death, one boy became the end—temporally and motivationally—of reproduction. Meanwhile, amid structural contexts of postcolonial modernization, biomedicalized reproduction, market transformation, economic aspiration, casteism, classism, and communalism, prospective parents increasingly avoided daughters as they began accumulating. During the 2010s, the sex ratio at birth for India's western region—essentially natural in firstborns—fell to 0.775 girls per boy after one daughter and 0.572 after two daughters (Kulkarni, 2020, 21). Affluence predicted sex selection, with wealth, education, caste privilege, and media exposure all correlating to more skewed child sex ratios (Arokiasamy & Goli, 2012; Kulkarni, 2020, 26–27; Visaria, 2007, 144–53). Despite extensive public health campaigning, “surpluses” of unmarried men, and some shifts in gender and kinship (Kaur & Kapoor, 2021, 120–23; Kaur et al., 2016), Gujarati families continued to choose against girls.

Champa‐ben's play did not delve into the complexities behind reproductive decisions. Instead, it invited us on a clearly mapped moral and sentimental journey. We frowned at the pregnant woman's distress. We snickered at the mother‐in‐law's bombast. During the climax, the doctor—cast as female despite 80% of local obstetricians being men—drew applause by thundering, “Don't you see? Son and daughter are equal! I'm a woman, and I'm a doctor! What if I'd been killed in my mother's womb? Don't discriminate between boy and girl!” The 2‐min denouement permitted Champa‐ben's character to reform. The cast then led us in a rousing chant of the official government slogan: “Save the Daughter! Celebrate the Daughter! Educate the Daughter!”

From character development to closing cry, Champa‐ben's play exemplified the “awareness” activities undertaken within a vast “reproductive governance” regime—a constellation of techniques by which state and nonstate forces sought to influence procreative behavior, simultaneously governing both bodies and moralities (Morgan & Roberts, 2012, 243; see also Gammeltoft, 2014, 29–76, 101–30, 228–34; Ginsburg & Rapp, 1995, 3–12; Mishtal, 2015; Singer, 2022, 11–16, 32–60). Alongside legal prohibition, sex selection had spawned widespread public health programming (Eklund & Purewal, 2017, 42–47). Government institutions were dominant purveyors, but mass media and nongovernmental organizations (NGOs) like Champa‐ben's women's association also participated.

Several weeks after the play, Champa‐ben and I were discussing her performance when her firstborn walked past. She nodded toward him: “God's mercy, we got a boy, first try. So, we didn't even have to consider all this. Otherwise, you *must*, and it's very tough.” Matter‐of‐factly, Champa‐ben recounted numerous acquaintances’ pursuit of sex selection. I gradually learned that most neighbors who had watched the play with me—most neighbors who had laughed, frowned, applauded, and shouted with me—had also condoned sex selection, if not undergone it themselves. All acknowledged gaps between the morality play we had witnessed and reproductive realities.

Such gaps offer windows on a key anthropological problem: how people respond to governance interventions against potentially harmful practices. Anthropological analysis and public health praxis often seem to assume that resonant messages can discipline individuals out of behaviors. But the mode of intervention exemplified by Champa‐ben's play—what I call “biopolitical melodrama”—shows how public health messaging can resonate without transforming dispositions or actions. Because moralized, affectively charged fantasies like the play partially misalign with social realities, they induce robust responses while failing to change behavior.

By illustrating how partial misalignments between story and sociality can manifest in the narrative governance of life, melodramatic anti‐sex selection messaging reveals subtleties in the operation of biopower. Biopower—a power to “*foster* life or *disallow* it”—is an integral part of modernity (Foucault, 1990, 138). It acts through dispersed processes of evaluation, normalization, and optimization that produce authoritative truths and then mold citizens and collectives accordingly; it therefore encompasses individual discipline, population regulation, and various links between the two (Foucault, 1984, 1990, 136–46). Among other effects, biopower's incitements to self‐manage can individualize responsibility, obscure structure, circumscribe discourse, and legitimize authority (Foucault, 1984, 1990, 141, 146–58; Stevenson, 2014).^5^

The biopower framework can help us understand governance of “unruly bodies and behaviors,” but applications sometimes proceed “as if a direct line between… biopolitical projects and individual experience and behavior were obvious and uncomplicated” (Rouse, 2021, 364, 362). Simplistic biopolitical analysis risks overdetermining dispositions and actions, leaving people little to do but conform or—more rarely—rebel (Reynolds‐Whyte, 2009, 11; Sleeboom‐Faulkner, 2010, 148–49). The challenge, then, is to explore people's participation in biopolitical governance while avoiding rigid determinism. Confronting biopolitical melodrama facilitates this by highlighting the values, stakes, interpretations, and ethical decisions that enliven biopower's truth claims and interventions (cf. Fassin, 2009, 48–49).

Combining the biopower framework with insights on morality, affect, and melodrama, this article examines what narratives do and do not achieve in Gujarati anti‐sex selection programming. I propose the concept of *biopolitical melodrama* to describe governance of potentially harmful behaviors through narratives featuring good‐evil binaries, stereotypical characters, emotional provocations, simplistic diagnoses, and triumphalist endpoints. In biopolitical melodrama, narratives assume the form, function, and content of biopolitical technologies. They convey authoritative, compelling truths about populations and bodies in ways ostensibly aimed at influencing order and action. Building on existing ethical and affective formations, melodramatic narratives furnish moral force and meaningfulness to interventions on life. Biopolitical melodrama engages people, and it can produce many classic biopolitical effects. But because it omits key social context, it can fail to impact behavior. Partial misalignment between melodramatic biopolitical narratives and lived realities may manifest in responses that neither fully oppose nor fully embrace governance interventions.

The quasi‐paradoxical coexistence of resonance and ineffectuality in Gujarati anti‐sex selection campaigns results from reliance on a melodramatic mode akin to that of popular Hindi cinema (cf. Nandy, 1981; Thomas, 1995). In public health, interventions often depend on narratives featuring stereotypical characters and plots (Briggs & Mantini‐Briggs, 2002; Vance, 2012); as explained below, when the interventions adopt a psychologized behavior change paradigm, the narratives easily veer toward melodrama. Broadly, the melodramatic mode responds to societal crises of truth and ethics (Brooks, 1995, 15; cf. Berlant, 2008). Amid such crises, political melodrama exhibiting five features—dichotomous morality, stock victim‐villain‐hero characterizations, sentimental provocations, facile diagnoses, and redemptive teleologies—can organize collective feelings of righteous struggle (Anker, 2014, 32–64; cf. Brooks, 1995, 11–12; Nandy, 1981; Thomas, 1995). Stitched together by synecdoche, decontextualization, and tangential association, political melodrama's features may activate strong, unambiguous feelings while erasing complexity (Brooks, 1995, 24–25; Vance, 2012, 203, 205; cf. Ang, 1996, 75–76). Like biopolitical interventions, melodramatic political narratives can offer authoritative truths, prescribe good citizenship, individualize structural problems, and legitimize power (Anker, 2014, 6–7, 20, 73, 111; Vance, 2012, 202; cf. Brooks, 1995, 203–4). Moreover, because political melodrama's resonance as an affective shorthand relies on congruence with existing moral formations (Brooks, 1995, viii, 5), it always operates within a “moral assemblage” (Zigon, 2011, 64) whose overlapping elements demand creative navigation.

Gujarati anti‐sex selection narratives packaged political melodrama in forms that resonated with parts—but only parts—of the local moral assemblage. Some instances, like Champa‐ben's play, unfolded as extended stories. Others, like posters and slogans, let spare images or sentences synecdochically represent entire fantasy templates. Either way, specious clarity emerged from casting endangered girls and population‐level balance as victims of backward villains. By simplifying the problem and positing a teleology of gender‐egalitarian resolution, melodramatic biopolitical narratives justified calls for individual transformation while ignoring structural contexts. A straightforward moral sentimentality legitimized institutions as women's protectors while displacing radical critiques. The messaging resonated for people. But it seldom changed behavior. As in Champa‐ben's play, biopolitical melodrama forged an ephemeral collective morality, inviting us to participate in the narrative governance of life without thereby making us its subjects.

In critiquing melodramatic narratives, I am not suggesting simplified health messaging is always pernicious. As a physician, I frequently use such messaging clinically. Yet, as a man committed to feminist praxis, I also believe justice requires questioning simplified representations when they impede understanding. As a US‐born and ‐raised Gujarati, I know we must avoid the essentialism plaguing much sex selection discourse. And yet, as child of a mixed‐caste but privileged couple, I also know we must not ignore the hierarchies that anti‐sex selection governance presumes and reiterates.

Below, I trace the rise of melodramatic anti‐sex selection messaging in Gujarat. I then analyze its three core ethical figures: the threatened daughter, the imbalanced population, and the backward family. In concluding, I consider biopolitical melodrama's implications for understanding people's relationships to the governance of life.

## “SAVE THE DAUGHTER”

**FIGURE 1:**
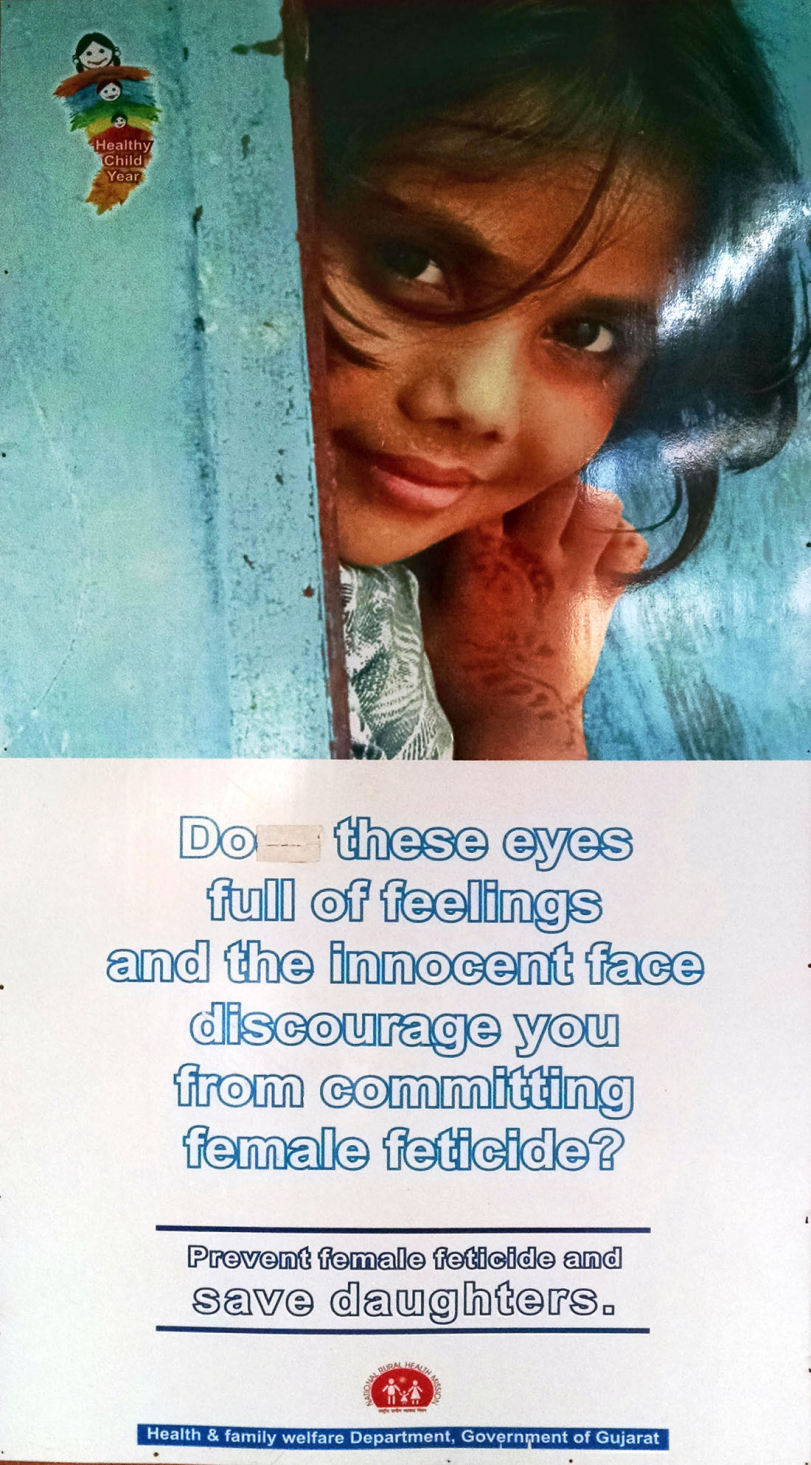
A floor‐to‐ceiling poster in the Gujarat health department's headquarters. The poster pairs an image of a girl with an anti‐sex selection message. (Utpal Sandesara/Gujarat Department of Health and Family Welfare) [This figure appears in color in the online issue]

Entering Gujarat's health department headquarters, I encountered a floor‐to‐ceiling poster (Figure 1). A giant visage hovered over dramatic words: “Do these eyes full of feelings and this innocent face discourage you from committing female feticide?”

It was early 2015. Since 2012, I had been conducting fieldwork that would eventually span 2000 h of direct immersion across clinics, households, and spaces of governance. For 11 months, I had spoken to dozens of doctors, nurses, and black‐market brokers while witnessing 172 sex determination scans and 29 selective abortions. During the same period, I had engaged with hundreds of families pursuing sex selection. Now, for 5 more months, I would immerse myself in the public representations and routine practices that make governance an everyday reality (Gupta, 2012, 42–74). I would take tea with government officials and attend public health events. I would speak with activists, community leaders, and citizens like Champa‐ben about behavior change messaging. And I would gather posters, brochures, and other media—the artifacts of biopolitical storytelling.

I gradually came to recognize both Champa‐ben's play and the giant threatened daughter poster as parts of a wider melodramatic regime. After originating as radical advocacy tactics, the regime's tropes had eventually become central to mainstream reproductive governance. They were contingent products of local, national, and transnational histories. But their development also reflected awareness‐oriented public health intervention's broader propensity for melodrama.

Prenatal sex selection itself initially emerged from mainstream biopolitical processes—processes that later clashed with Save the Daughter biopolitics. Population control and family planning are *the* organizing principles of India's postcolonial reproductive governance (Rao, 2004; Sreenivas, 2021). Legalization of abortion in 1971 was largely a response to domestic and international pressure for reduced fertility (Rao, 2004, 19–74; Roy, 2023, 203–7; Sreenivas, 2021, 124–65). Four years later, India's first report of amniocentesis touted prenatal sex selection as a potential boon to population control: “In India cultural and economic factors make the parents desire a son, and in many instances the couple keeps on reproducing just to have a son. Prenatal determination of sex would put an end to this unnecessary fecundity” (Verma et al., 1975, 384). As amniocentesis and ultrasound proliferated in the 1980s and 1990s amid state policies that favored family planning, biomedicalized reproduction, and a transnational economy of technology, sex‐selective abortion mushroomed.^6^

Early on, agitation against sex selection was a radical effort. Feminist organizers mounted grassroots campaigns to raise awareness and advocate change. While contextualizing sex selection vis‐a‐vis broader patriarchal injustices, these early campaigns also used shock tactics like fetal personification, bloody imagery, and the language of “female foeticide” (Gupte, 2003).

When Parliament outlawed prenatal sex determination in 1994 (leaving abortion law untouched), the debate centered the nation's future as much as gender justice (Roy, 2023, 207–12). Biopolitics had run amok, jeopardizing the state's legitimacy as guarantor of order and equality. The brakes were finally being applied. Nonetheless, 2001 census data showed widespread worsening of gender skewing, and advocacy efforts gained fresh urgency.

A judicialization of fetal biopolitics (cf. Biehl, 2013) finally pushed melodrama into the mainstream. On May 4, 2001, the Supreme Court ruled on public‐interest litigation by feminist activists.^7^ The judgment mandated stronger enforcement of legal measures. But it also prescribed a new governmental intervention: “awareness campaigns.” By valorizing “awareness,” the Supreme Court invoked a psychologized approach, long axiomatic in global public health, that posits modification of knowledge and attitudes as central to behavior change (Bettinghaus, 1986; Briggs & Mantini‐Briggs, 2002, 35, 112). In classic biopolitical fashion, the knowledge‐attitude‐behavior approach calls for enlightening and empowering individuals according to authoritative truths about health‐related practices. Because the paradigm emphasizes attitudinal change, it encourages ethical and sentimental education alongside information delivery. In early‐2000s India, it fostered melodramatic anti‐sex selection governance.

Multiple contemporaneous influences shaped the biopolitical melodrama that entered Gujarat's spotlight. Globally, anti‐sex selection awareness campaigns fit into a turn‐of‐the‐millennium shift toward interventions prioritizing “the girl child”—a shift that reconfigured care of girls as the panacea for various political, economic, and moral ills (Murphy, 2017, 113–24), priming government officials, journalists, activists, and researchers to attend to “missing women.” Nationally, awareness campaigns entered the wider milieu of India's early‐2000s reproductive governance, which rearticulated postcolonial nationalism, patriarchy, classism, casteism, communalism, and population alarmism while artificially isolating individual “consumers” of medical technology (Rao, 2004, 23, 181, 196, 268; Sreenivas, 2021). And locally, biopolitical melodrama's rise paralleled Gujarat's embodiment of a specific Indian modernity—a “Gujarat model” steeped in Hindu nationalism, economic liberalization, and peculiar ambivalences toward violence (Ghassem‐Fachandi, 2012; Sud, 2022). Alongside kinship norms and socioeconomic aspiration, these structural processes constituted the moral assemblage that mainstream melodramatic messaging—dedicated to girls, grounded in a patriarchy it explicitly abjured, and molded to the modern Gujarati psyche—came to join.

Following the 2001 Supreme Court judgment, the NGO CHETNA spearheaded Gujarati awareness efforts. It collaborated on advocacy trainings, seminars for public officials, communication campaigns, and media outreach to “mobilise public opinion for ensuring the health and dignity of the girl child” (Vakharia, 2009, 32). In conjunction with the United Nations Population Fund and the Population Foundation of India, CHETNA launched a “Missing Girl” program around Mahesana to “sensitise caste leaders/youth and couples” (Vakharia, 2009, 31–32).

**FIGURE 2:**
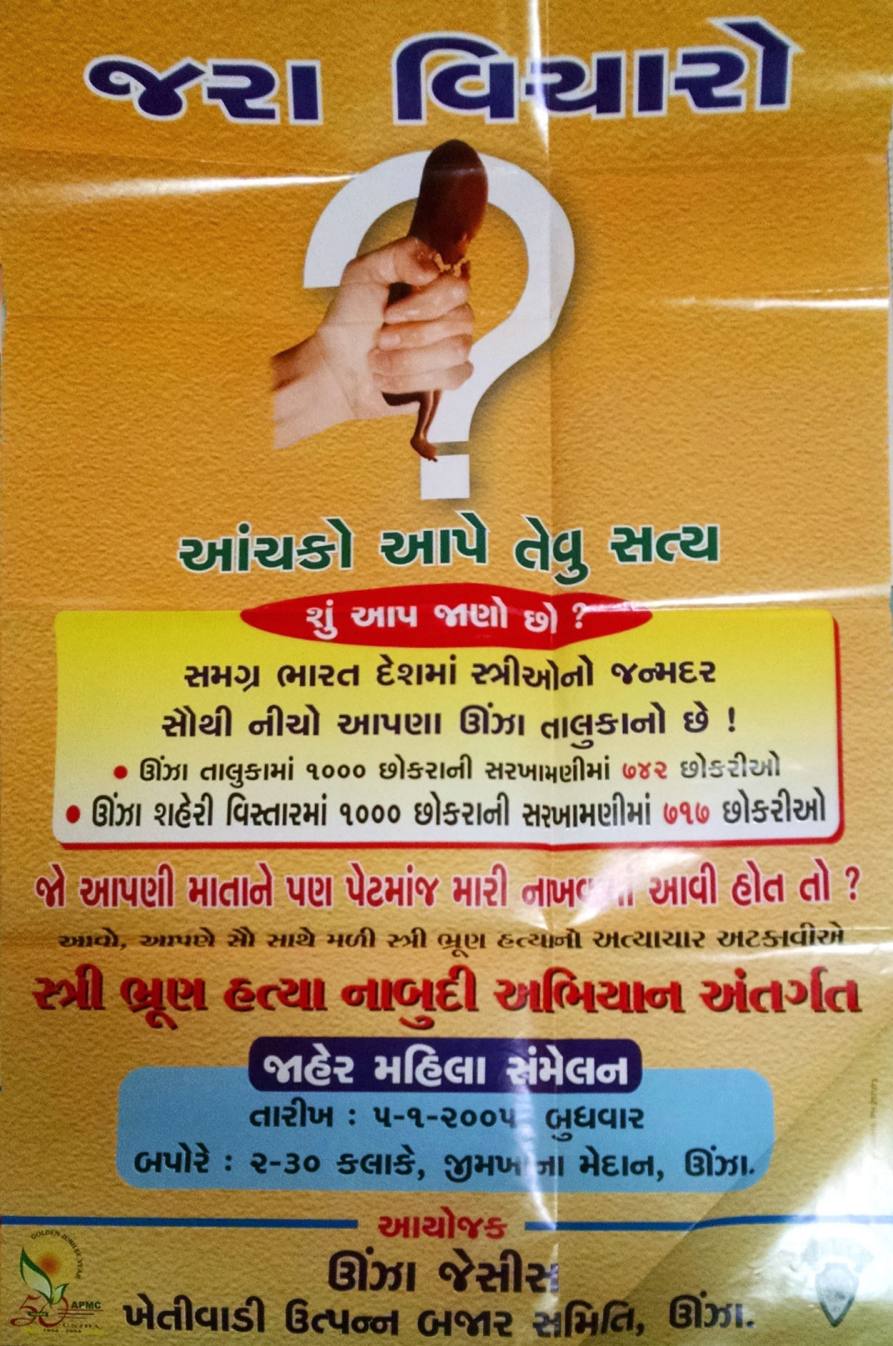
An advertisement for the public assembly “Social Awareness and Elimination of Female Foeticide,” held in Unjha on January 5, 2005. The title and subtitle read, “Think about It: A Truth that Will Jolt.” (Utpal Sandesara/Unjha Jaycees) [This figure appears in color in the online issue]

Inspired by CHETNA's work, the parliamentarian representing Mahesana District's Unjha constituency partnered with community leaders to stage an assembly that augured and inspired widespread dissemination of anti‐sex selection melodrama. Advance publicity for the event, billed as “Social Awareness and Elimination of Female Foeticide,” already foregrounded moralistic sentimentality. Invitations featured a “Respectfully Addressed Letter to a Cruel Mother‐Father” from “an aborted daughter‐fetus growing in the womb”; the imagined girl lamented broken dreams at length before concluding, “*I hope no one gets a mother‐father like you*.” A public advertisement (Figure 2) juxtaposed a fetus‐crushing fist with local ratios of girls per 1000 boys: 742 for Unjha Subdistrict, 717 for Unjha city.

**FIGURE 3:**
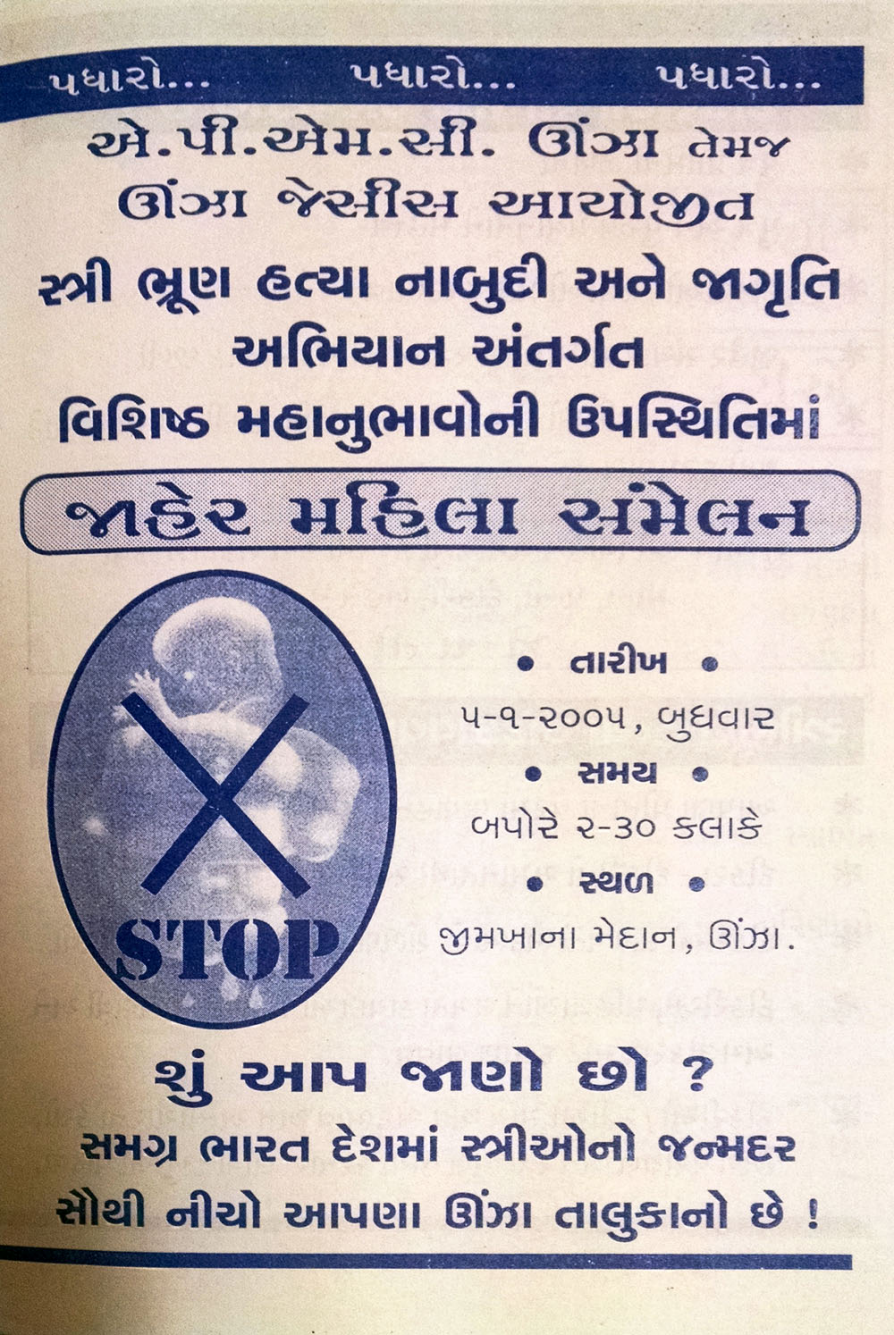
A pamphlet distributed to the attendees of the 2005 Unjha assembly “Social Awareness and Elimination of Female Foeticide.” The cover features a picture of a fetus‐crushing fist, captioned by the word “STOP.” (Utpal Sandesara/Unjha Jaycees) [This figure appears in color in the online issue]

On January 5, 2005, thousands of women filled a vast gymnasium, accompanied by Unjha's obstetricians. Speakers condemned gender inequality. Dignitaries lit lamps symbolizing commitment to girls. Everyone recited oaths against “female foeticide.” Attendees received a booklet highlighting Unjha's skewed girl–boy ratios and the projected consequences of population imbalance (Figure 3). A second booklet, “Eyewitness Report of a Child's Murder in the Womb,” refashioned the US anti‐abortion film *The Silent Scream* into a grisly account of sex‐selective abortion. Both booklets replicated the fetus‐crushing fist.

The Unjha assembly was an early, archetypal example of biopolitical melodrama's reliance on oversimplifications, victim–villain–hero characterizations, and emotional provocations. These elements soon permeated Gujarati public health efforts. Moreover, given the Unjha event's scale, political backing, and straightforward message, it engendered mimicry across India, contributing to nationwide diffusion of melodramatic tropes (CYDA, 2007, 199, 221–22, 228).

Even as melodramatic messaging spread, sex selection remained pervasive. The 2011 Census showed many states and localities, including Unjha, with stagnant or worsening child sex ratios. Mahesana‐area families continued seeking sex selection. Local doctors continued offering it. It is tempting to conclude people engaged in a “politics of duplicity,” falsely professing conformity with a public narrative while maintaining private commitments to different truths and motivations (Kligman, 1998, 13–15).

But melodramatic narratives did not ring false. I discussed the Unjha event years later with multiple attendees. All had continued pursuing, condoning, or performing sex selection. But their reminiscences showed melodramatic messaging's ethical and affective resonance. Eyes welled up. Voices trembled. Phrases like “a tremendous event” and “heartbreaking, tear‐jerking words” resounded in awed tones. We cannot discount some performance for me. But the spontaneity, ubiquity, and consistency of people's reactions indicated a relevant biopolitical limit. Anti‐sex selection messaging produced a shared (if evanescent) morality. Sentimentalized portrayals of an idealized good (even if only fantastical) enlivened an energetic (if ultimately specious) sense of doing *something*. Alignment between the Unjha event's underlying narrative and sociocultural reality was sufficient to elicit strong feelings, though insufficient to transform behavior.

In subsequent years, melodrama transformed from radical tactic to dominant technology. Indian feminists had begun reconsidering melodramatic tropes, which they realized were susceptible to decontextualized, regressively patriarchal interpretations; some already regretted “evangelizing through any means possible” (Gupte, 2003). But melodrama was swiftly becoming the moral and affective core of mainstream anti‐sex selection governance. On International Women's Day 2006, Gujarat Chief Minister Narendra Modi proclaimed a statewide campaign to “Save the Daughter.” People started invoking the slogan in political rallies and sermons, motorcycle parades and marriages. A decade later, I found Mahesana awash in melodramatic anti‐sex selection messages. The state government was a major purveyor, but there were many. Most local advocacy, journalism, and community programming used the same language of “Save the Daughter” and “female foeticide,” and most recapitulated the same narrative about threatened daughters, imbalanced populations, and backward families.

Prospective parents usually found the narrative resonant. But resonance discouraged relatively few. Even those deterred by governance messaging acknowledged the messaging's incongruence with everyday realities of kinship, economic rationality, and fertility control. And those deterred were ultimately exceptions punctuating a broader pattern: resonance without transformation, participation without conversion.

## THE THREATENED DAUGHTER

**FIGURE 4:**
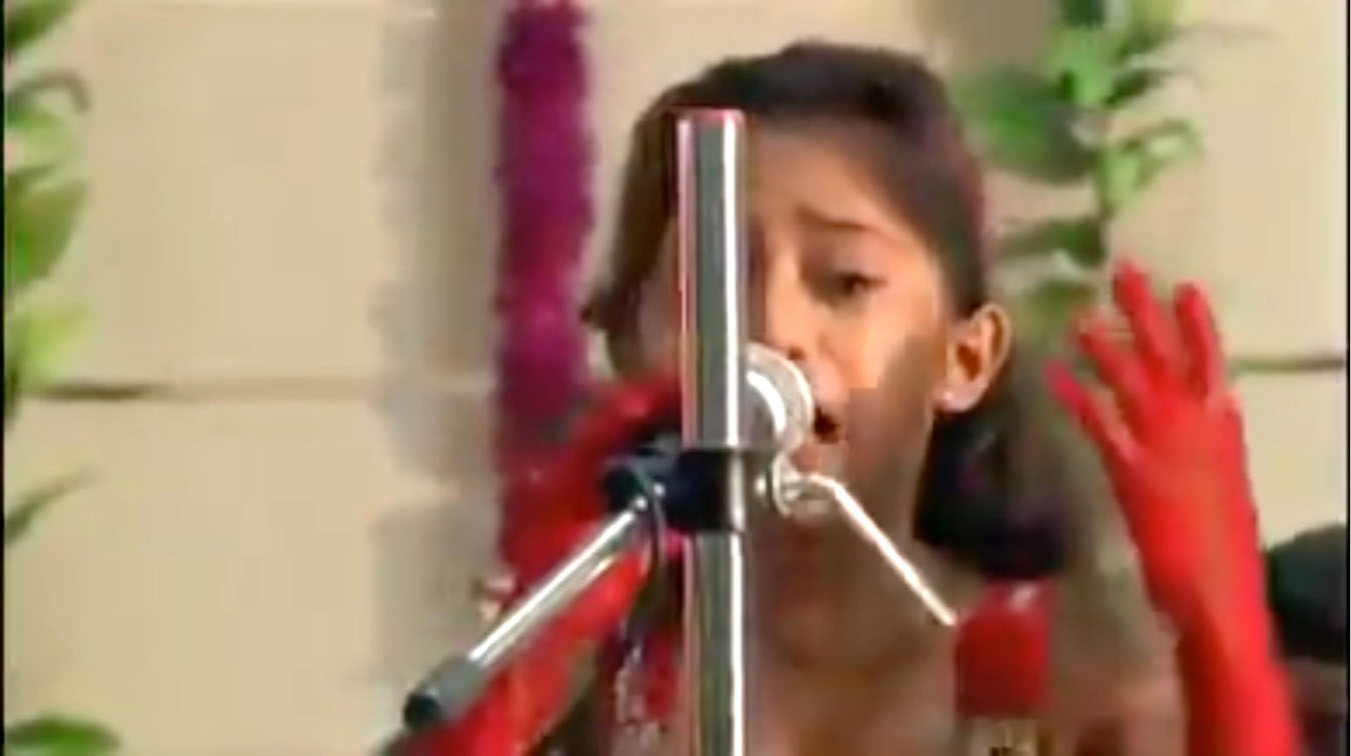
A still from a video that circulated widely on Gujarati social media in 2014. In the video, a young girl denounces sex‐selective abortion. (YouTube) [This figure appears in color in the online issue]

During the 2014 monsoon, everyone wanted to share the same viral video. Again and again, I watched a grade‐schooler deliver a fiery speech (Figure 4). Tiny hands gripping a microphone, she began, “My topic today is female foeticide. Today, I'll tell you an unborn daughter's anguish – a letter from an unborn daughter to her mother.”^8^

The nine‐minute performance recurrently appealed to love and guilt:

Oh, Ma! I wanted to come see the beautiful world, to know it. But you, for your own happiness, shattered all my hopes…. Oh, my tenderhearted Ma, how can you do this to me? How can you bear to butcher the delicate body of the dear little daughter growing in your womb?…

The girl's descriptions of sex‐selective abortion rivaled the most graphic US anti‐choice propaganda.

Midway, the speech leaned heavily into patriarchal kinship norms:

Watch, Ma. I'll have… wedding music playing, and I'll be like a bird of your garden and fly away. Don't make me fly away prematurely….

A daughter ties everyone—grandparents, parents, brothers—with a bond of love, and that's why everyone weeps when she goes to her conjugal home. A daughter is a father's world, a mother's support, a brother's love….

In closing, the girl sang a song punctuated by mimed tears.

Couples eagerly shared the video while waiting for clandestine ultrasounds and abortions. The moralistic, sentimental performance moved them. Many framed it similarly: *This shows why what we're doing is wrong*. Nonetheless, they deemed sex selection inescapable—a necessary evil.

*

From pint‐sized orators to gigantic posters, the threatened daughter figure resonated. Anti‐sex selection messaging constructed victimhood narratives around a female “fetal citizen” (cf. Casper & Morgan, 2004; Holc, 2004)—a prenatal “girl child” worth saving and valuing (cf. Murphy, 2017, 114–25). These narratives exemplified how humanitarian governance can make rescue imperative by casting precarious lives as abject, innocent figures deserving pity, representation, and assistance (Fassin, 2012, x, 1–4, 7–8; Kapur, 2002; Vance, 2012). More concretely, threatened daughter narratives exemplified how spectacles of vulnerable bodies can communicate putatively self‐evident truths about violated rights (Allen, 2009; Fassin, 2012, 161–80; cf. Anker, 2014, 49–50; Brooks, 1995, 56–80). Leveraging personification and ventriloquy,^9^ spectacles of the threatened daughter mobilized moral sentiments around female victimhood, stirring up an affective mix all too familiar from girl‐centered campaigns worldwide: “anxiety about… economic futures; empathy for the harsh and cruel lives of some girls; attachments to broken girls known or imagined; hope and admiration for the amazingness of girls; liberal feminist aspirations for agentic and equal life; [and] personal desires to help and rescue” (Murphy, 2017, 121–22).

**FIGURE 5:**
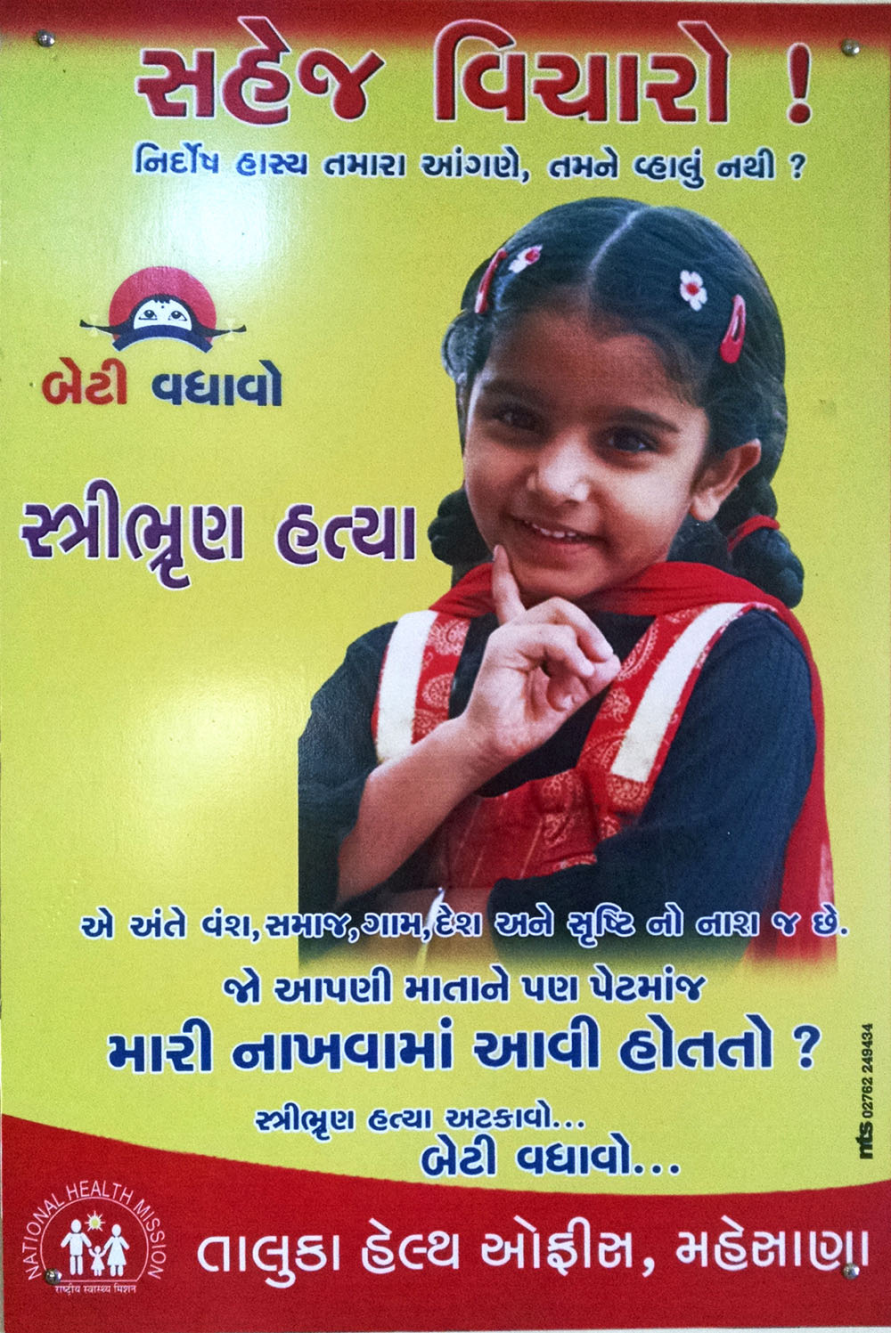
A Gujarat health department poster. The poster pairs an image of a preciously posed girl with the words, “Stop and think! Isn't innocent laughter on your doorstep dear to you?” (Utpal Sandesara/Gujarat Department of Health and Family Welfare) [This figure appears in color in the online issue]

To conjure victimhood, public health materials paired tender visuals and dramatic verbiage. In the viral video, dainty appearance and mimed tears synergized with shocking words. The giant threatened daughter (Figure 1), with “eyes full of feeling” and “innocent face,” operated similarly. Another government poster (Figure 5) juxtaposed a smiling girl with the lines, “Stop and think! Isn't innocent laughter on your doorstep dear to you?” Emotional appeals moved potential daughters from liminality to personhood, incarnating them as full‐fledged victims needing rescue—as foci for “a bloom of possibility, a bouquet of potential, a cluster of affect” (Murphy, 2017, 120).

**FIGURE 6:**
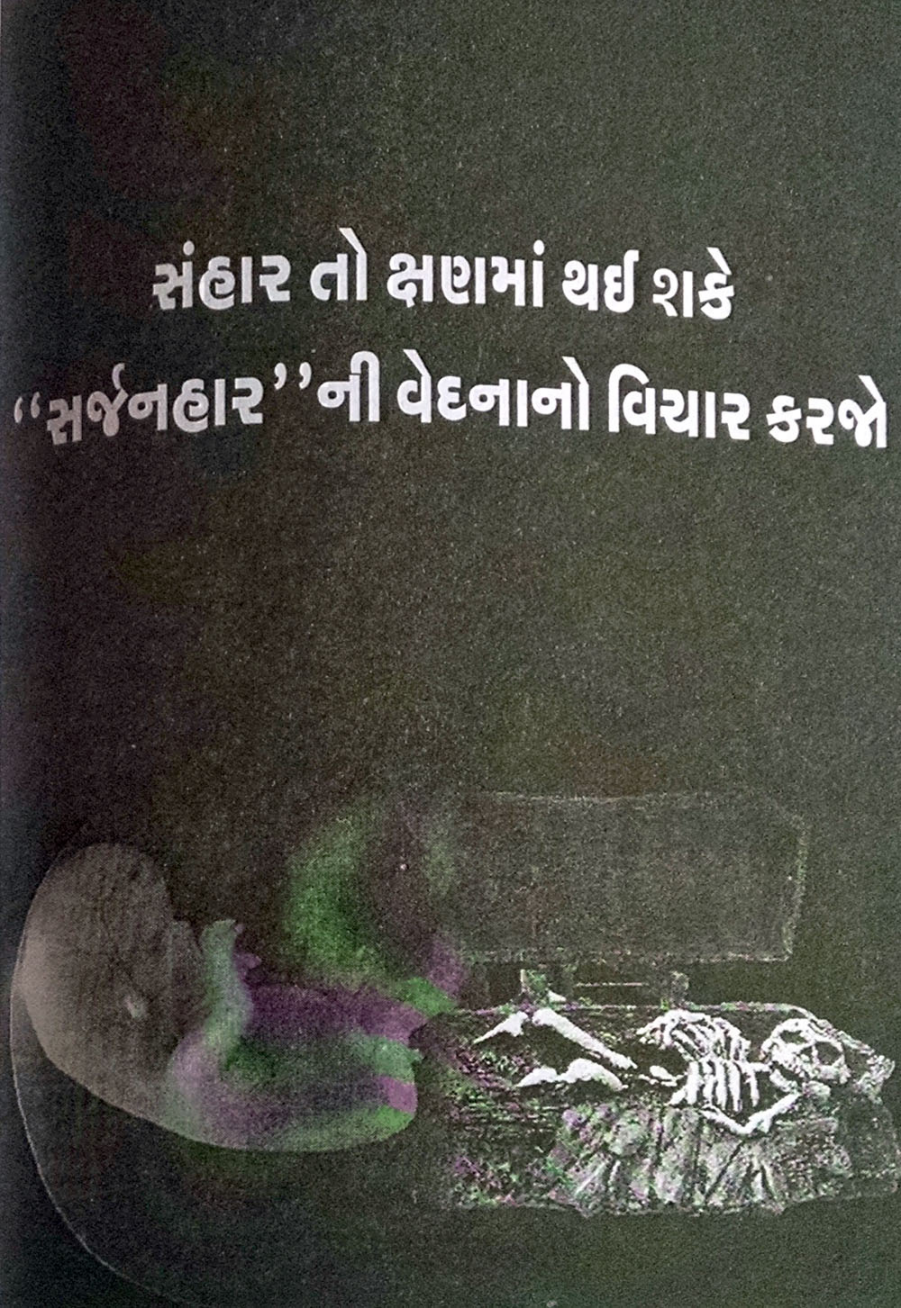
An illustration from an activist's anti‐sex selection book. The picture juxtaposes a fetal silhouette with a skeleton in a coffin. (Rashmi Hada) [This figure appears in color in the online issue]

**FIGURE 7:**
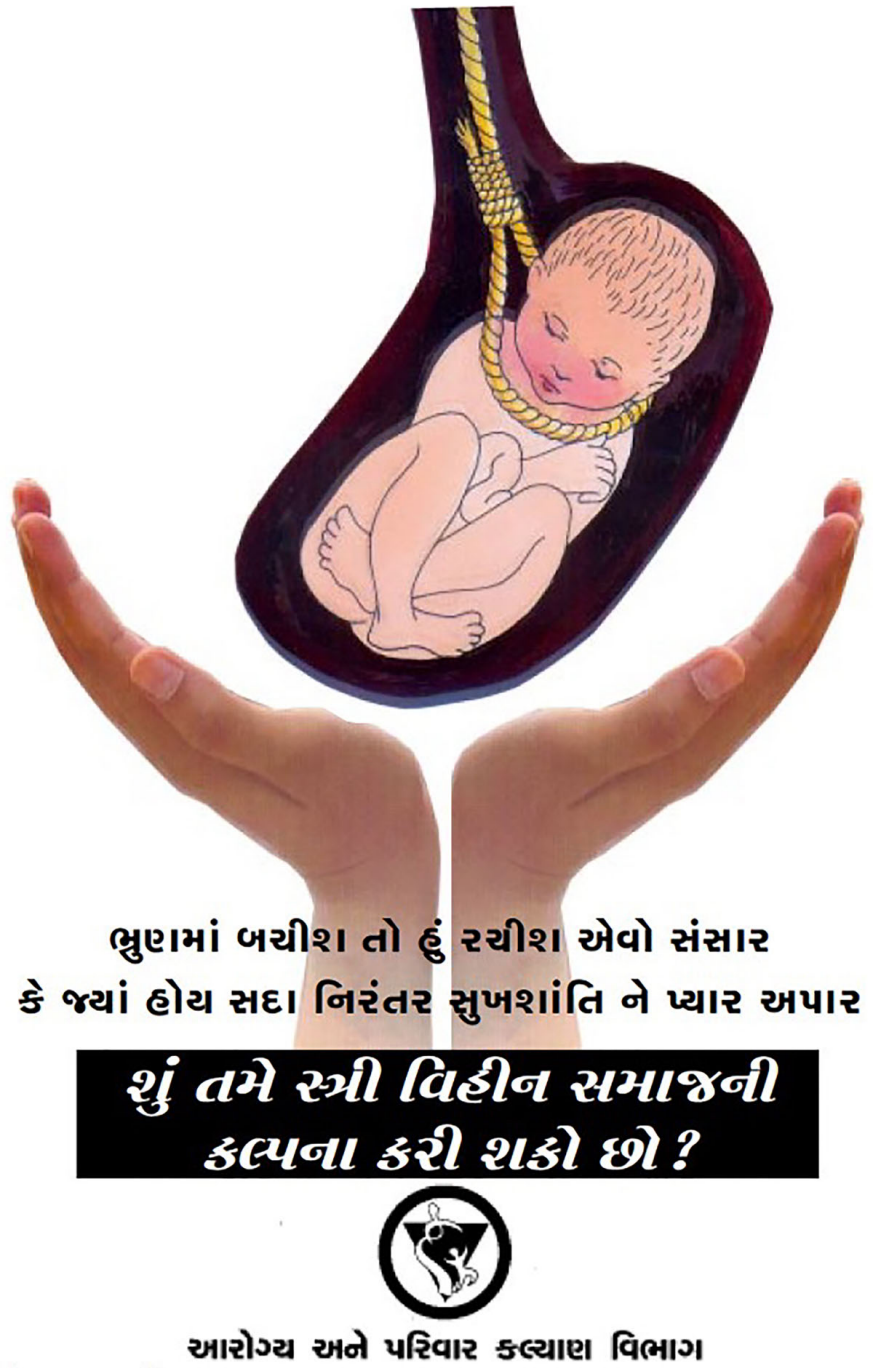
A Gujarat health department poster. The poster depicts a noose around an in utero infant and asks, “Can you imagine a world without girls?” (Utpal Sandesara/Gujarat Department of Health and Family Welfare) [This figure appears in color in the online issue]

**FIGURE 8:**
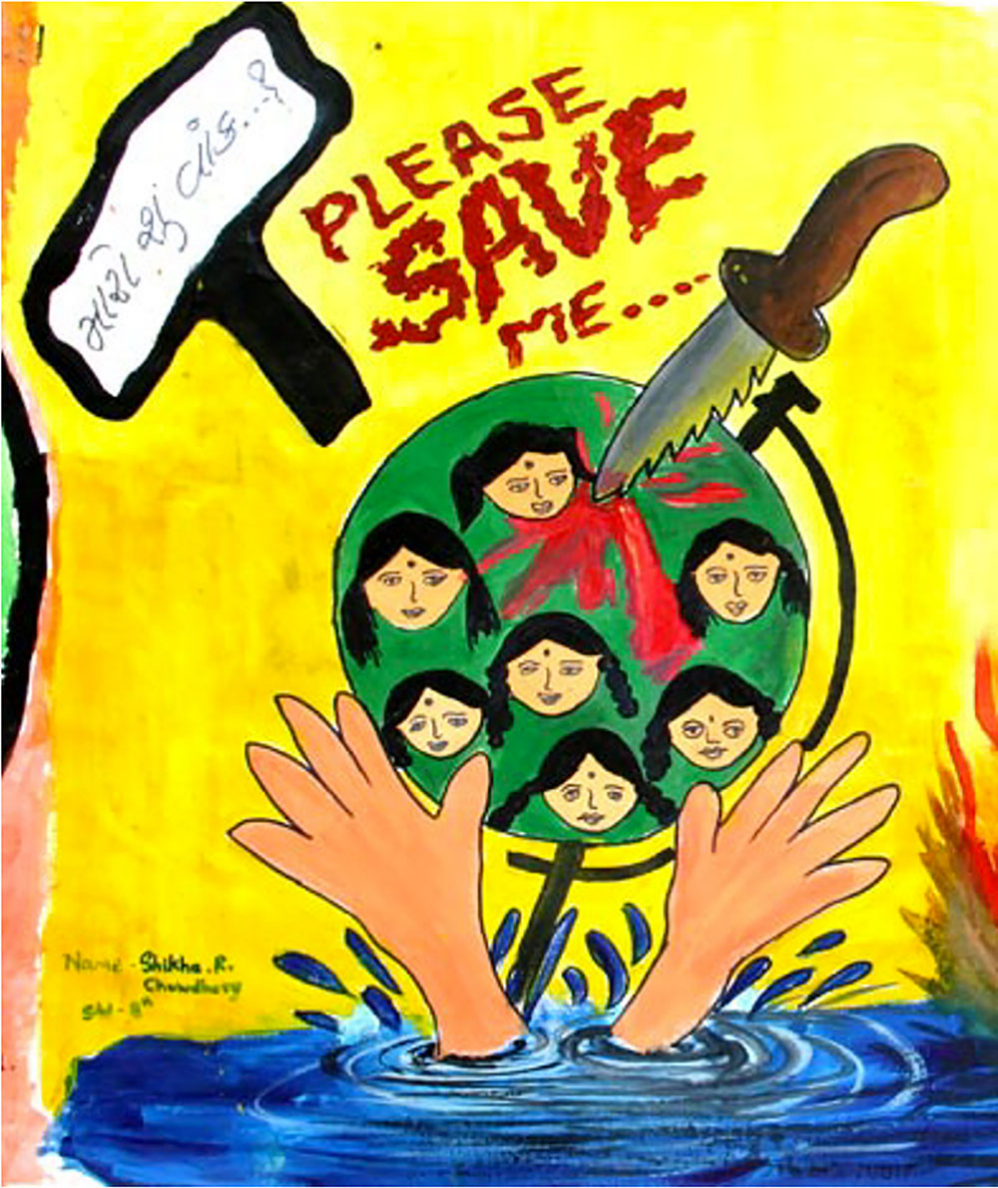
A schoolchild's artwork as reprinted in an anti‐sex selection book. The image depicts a knife stabbing a globe full of girls; the words above read, “What is my fault…? PLEASE SAVE ME…” (Shikha Chaudhary/Rashmi Hada) [This figure appears in color in the online issue]

Building on fetal personification, public health messaging portrayed sex‐selective abortion as murderous and sinful, despite the obvious contradictions with permissive abortion laws and antinatalist biopolitics (cf. Roy, 2023, 209–12). Materials emphasized daughters’ “right to be born.” Grotesquerie overflowed: fetal silhouettes alongside skeletons in activist art (Figure 6), nooses strangling in utero infants on government posters (Figure 7), knives stabbing girls in school art projects (Figure 8). Even though abortion was legal, widely accepted, and actively promoted elsewhere, anti‐sex selection messaging repeatedly invoked “foeticide.” In “vibrant vegetarian Gujarat” (Ghassem‐Fachandi, 2012, 153–212), such denunciations of violence touched a particular nerve.

Despite moral and sentimental appeal, victimization narratives did not challenge fundamental inequalities. In Orwellian fashion, billboards, slogans, and productions like Champa‐ben's play performatively negated lived realities through facile statements: *Sons and Daughters are Equal!* Sometimes, melodrama's reliance on existing ethical formations meant threatened daughter narratives directly *reinforced* patriarchal kinship. Materials such as the viral video emphasized women's usefulness to men: joy for fathers, ritual fulfillment for brothers, matrimony for husbands. Like the viral video, programming frequently reminded audiences of the daughter's inevitable out‐marriage, awkwardly framing the sorrow of separation as a sign of value.

Threatened daughter melodrama resonated for Mahesana‐area couples. It aligned with their convictions, feelings, and lived experiences parenting girls, which most did with affection, pride, and concern. Felt resonance, in turn, legitimized institutional efforts. “What Narendra Modi says is right,” couples pursuing sex selection told me. “We *should* be saving girls.”

But threatened daughter melodrama did not confront the structural realities that made one boy necessary and additional girls unacceptable—a stark illustration of the way structural factors are “not so easily depicted, understood, or confronted as causal agents” in the melodramatic mode (Vance, 2012, 202). Narratives of the threatened daughter did not overturn gender‐kinship norms, decelerate land consolidation and market expansion, or tame economic and educational aspirations. Consequently, such narratives did not necessarily transform practices.

Govind‐bhai, a prosperous land broker, illustrated this dynamic. I met him and his wife when they sought contraception to facilitate a “break” between three successive daughters and the inevitable son. Govind‐bhai adored his girls. He obsessively tracked the infant's weight. He boasted about the grade‐schooler's academic achievements. He horseplayed with all three and performed countless banal acts of care. He and his wife had accepted more daughters than many couples, and he considered the goal of saving girls logical and legitimate.^10^ Pointing to his youngest, he said, “If you get it taken out, that's a sin! Killing a human? That's the greatest killing… If she so much as cries, it burns my heart.”

But Govind‐bhai and his wife were also determined to pursue sex selection in the next pregnancy. “At this point, it's all for a boy…” he told me. “We're going to get it checked, final. We've reached the position where sin seems right – if we must do it, we'll feel we must do it. It's a sin, but it's a necessary sin.” He rattled off the pragmatic considerations—cost and care, support and loss—that made sex selection commonsensical.

Similarly, multiple couples opting for selective abortion explained their decision using a passage from a Hindu sermon widely circulated in NGOs’ anti‐sex selection campaigns:

Daughter is compassion's epitome. She leaves behind affection and goes to her husband's home.… A daughter endures all of life's incidents by her own politeness and the virtues her parents have inculcated. She suffers them….

The son is his father's hand, but the daughter is his heart. Indeed, that's why, when a father gives a daughter's hand in the son‐in‐law's hand… he's giving away his heart itself.

The passage brought tears to parents’ eyes. But where public health campaigns framed its poignancy as proving girls’ value, families emphasized the suffering imposed on daughters and their parents. People inhabited governance narratives and nonetheless ‐ or perhaps, therefore—insisted on sex selection.

## THE IMBALANCED POPULATION

In early 2015, I accompanied government official Hitesh‐bhai to a public health seminar. Lounging behind a table, he thundered down at women seated in a dusty village plaza.

“In 2014, only 17 girls were born against 39 boys in your village!”

After railing against presumed motives for sex selection, Hitesh‐bhai continued, “I'll give you an anecdote. In my village, 17 men couldn't find wives in their [caste] communities!” The men, he explained, ended up marrying women from eastern Gujarat's tribal groups. The audience gasped and tut‐tutted.

Describing the new brides’ dress and etiquette, Hitesh‐bhai further baited anxieties about social change:

Beware! Our *sanskār* [mores, manners] are changing! Now, daughters‐in‐law sit on chairs and make fathers‐in‐law sit on the ground. Now, you can't even criticize daughters‐in‐law!…

Why'd the neighborhood's *sanskār* go bad? Because they brought girls from outside. And why'd they bring from outside? Because they couldn't find anyone in the community. And why couldn't they find anyone? Because we didn't let daughters be born! Remember this!

Hitesh‐bhai offered another cautionary tale about gender imbalance and intermarriage, this one revolving around loosening of the oppressive taboo against inter‐caste commensality. The audience obliged with frowns and groans.

Hitesh‐bhai then sketched out a dystopian future: “If boys don't find girls, they'll kidnap our daughters…. You must know such cases are rising. In the future, if girls are fewer than boys, rape and crime and all that'll increase so much.”

The event ended with recitation of the “Save the Daughter” slogan.

*

From the 2005 Unjha assembly to Hitesh‐bhai's seminar, the imbalanced population pervaded Gujarati public health messaging. Renderings were statistical and impressionistic, large‐scale and local. Regardless of form, the figure of imbalance fulfilled two important functions. First, as in the Unjha materials, it was a (quasi‐)quantitative “technology of truth” (Merry & Coutin, 2014), measuring up threats to precarious life. But as in the original parliamentary debate on sex selection (Roy, 2023, 210–12), the imbalanced population also communicated disruptions to normative living (cf. Brooks, 1995, 20). Depictions of skewing became devices for eliciting anxieties around social change, and for channeling those anxieties toward the project of rebalancing. Paralleling other girl‐centered campaigns globally, the figure of population imbalance articulated “speculative futures about girls that [could] take the form of a dread of diminishment or an excited premonition of a better life” (Murphy, 2017, 113–14).

Reflecting motherhood's centrality in propagating postcolonial Indian identities (Sreenivas, 2021, 166–201), public health messaging framed imbalance as a threat to social reproduction. The “innocent laughter” poster (Figure 5) declared: “Female foeticide: ultimately, it's the destruction of lineage, community, village, country, and all creation!” Some materials explicitly invoked patriarcho‐nationalism (cf. Mayer, 2000), as in a government poster pairing a feminine face with masculine patriots’ names: “I could be mother to a future Gandhi, Sardar, Tagore, or Bhagat Singh waiting in the womb.” Creative works with titles like “Anguish of the Unmarried,” voiced through imagined bachelors, lamented shortages of brides. Men, lineages, and the patriarchal order became the real victims of sex selection, with daughters figuring chiefly as daughters‐in‐law, wives, and mothers.

Fear and pity around threatened social reproduction dovetailed with dread, disgust, and outrage about caste mixing and misbehaved brides. As in Hitesh‐bhai's parables, anti‐sex selection programming frequently hijacked regressive gender and caste biases. Citizens could construct sinister subtexts around bland statements about men not finding wives “in their communities.”

Anxiety‐mongering peaked when imbalance narratives projected violent futures. Messaging warned that gender skewing portended rampant rape, kidnapping, and harassment, leveraging classism by playing on the widespread common sense (often unstated) that men remaining unmarried were disproportionately poor, uneducated, and rural‐dwelling. Again, saving girls was about satisfying men, who would otherwise have “no choice,” as many put it, but to violate sexual boundaries.

Melodramas of imbalance moved people. Like Hitesh‐bhai's audience, couples pursuing sex selection frowned at figures, decried intermarriage, and fretted about futures. Amid Gujarat's communalization, many Hindus also linked gender skewing to the specious prospect of minoritization due to Muslim “over‐reproduction.”

But melodramas of imbalance did not necessarily discipline behavior, for they failed to address structural contexts and household motives. The tacit biopolitical imperative in narratives of imbalance was heroic martyrdom—self‐sacrificially parenting multiple girls to improve abstract numbers. This imperative clashed with the simultaneous biopolitical imperative to limit fertility, and with everyday realities of parenting amid volatile socioeconomic conditions and relatively static kinship practices. People widely perceived more daughters to mean more hardship, a perception the state and NGOs constantly reinforced through family planning messaging. Despite anxieties about social disruption, couples generally would not subordinate family balance (and small families) to population balance.

A Mahesana‐area government nurse told me:

People understand: *My son won't marry by my having a daughter!* Once, a beneficiary wanted a ‘check.’ She was uneducated, so I couldn't talk about ratio. Instead, I said, ‘Ben, if we don't keep girls, boys'll be left unmarried.’

She said, ‘Ben, how's my son going to marry by my having a daughter? My daughter'll let someone *else's* son marry! Why should I keep birthing girls for someone else?’…

We can explain ratios—that the government's trying to preserve social balance. But everyone'll say the same thing: “No more girls in my house. To preserve balance, let them be born at my neighbor's!”

Couples pursuing sex selection corroborated the nurse's observations. Biopolitical rationalities and household rationales were misaligned. Imbalance messaging resonated, but without challenging sex selection's commonsensicality.

Dystopian narratives went even further, reinforcing aversion to girls. Why birth more, couples asked, when raising them was so fraught? Some cited infamous sexual assault cases. Others revoiced moral panics about elopement and “love jihad,” the apocryphal practice of Muslim men seducing and converting Hindu women. Worries about sexual violation and transgression underscored how imbalance narratives perversely reiterated the patriarchal norms of purity and propriety that helped drive sex selection.

## THE BACKWARD FAMILY

Who ultimately bore blame in Gujarat's anti‐sex selection melodramas? Who was the fetus‐crushing fist, the wielder of noose and knife, the perpetrator of “female foeticide,” the unfeeling killer?

In a self‐referential twist, Gujarati public health narratives interpellated audiences into the plot as both villains and heroes. As villains, citizens embodied ignorant, misogynistic backwardness. The diagnosis of backwardness justified education designed to transform erstwhile villains into heroes. A triumphalist teleology emerged, shifting blame for persistent sex selection onto individuals while obscuring structure. Just as global “invest in a girl” campaigns hold out the allure of “the right lever to be pulled, the right button to push, the correct commodity to purchase that will fix the world without having to undo capitalism itself” (Murphy, 2017, 121–22), anti‐sex selection messaging promised that the “right” reproductive choice would fix the world without having to undo patriarchy itself. The backward family became an alibi (cf. Murphy, 2017, 122) for contemporary Gujarat's political and moral economies of gender.

Governance interventions framed sex selection as “irrational” reproduction (Krause & De Zordo, 2012) rooted in psychologized backwardness. While “tradition,” “culture,” and “society” all featured prominently in messaging, these became problematic chiefly through people's “mentalities” and “preferences,” as when a CHETNA retrospective attributed skewed child sex ratios to “age‐old mindset/discrimination toward the girl child and strong son preference” (Vakharia, 2009, 30). Personifications of backwardness abounded. Champa‐ben's performance lampooned narrow‐minded mothers‐in‐law. Government posters, the Unjha advertisement, and the viral video excoriated prospective parents for ignorance and callousness. Messaging divided people into modern and antimodern, progressive and regressive (cf. Briggs & Mantini‐Briggs, 2002, 33–36). Anti‐sex selection campaigns recapitulated colonial campaigns against female infanticide and sati—attempts to modernize the “savage family” (Sen, 2002) or “contentious tradition” (Mani, 1998) under the pretext of rescuing female victims.

With backwardness the diagnosis, awareness‐oriented education became the logical solution. From the viral video to the doctor's diatribe in Champa‐ben's play, biopolitical messaging exhorted people to move from ignorance to enlightenment. As in the Unjha event's title, “social awareness” and “elimination of female foeticide” became inseparable.

The goal of awareness authorized a pedagogy rooted in the knowledge‐attitude‐behavior paradigm. Governmental efforts fell under the Gujarat health department's “Information, Education, Communication” program, which presupposed that “refuting myths and understandings” would transform attitudes and actions (National Health Mission, 2015). NGOs operated on similar assumptions, as one activist explained:

It's a standard approach: if you give knowledge, attitudes change, and that changes behavior. So, if I have a conversation with them, give them information, then they'll change their thinking, and perhaps put that into action. Now, knowledge includes information, like the 0–6 sex ratio. It also includes matters that touch the heart. So, the other person gets sensitized… Knowledge makes individuals so empowered, they can solve their own problem…

The narrative of transformation presumed transformation through narrative. Beholding some unheeded indicator of truth would enlighten people, empowering them to rescue rather than reject daughters—to become responsible modern citizens.

Incitements to overcome backwardness thus shaped an individualizing teleology of redemption. Governance messaging devolved responsibility onto citizens, detaching choices and practices from the contexts that gave them meaning. Solutions to sex selection would emerge not from radical transformation of the conditions that made sons indispensable and daughters unwanted, but from the “juxtapolitical” activity of people addressing structural problems through intimate fixes (Berlant, 2008, 10). Stock characters like Champa‐ben's swung rapidly from antimodern villainy to progressive heroism. The implication? Audience members could, too. In another self‐referential twist, the triumphalist plot carried a built‐in excuse: if the threatened daughter was not saved, it was only because backward citizens had not yet redeemed themselves.

Women disproportionately bore the burden of redemption. Like Champa‐ben's play, much messaging singled out mothers‐in‐law. Potential mothers faced even more vitriol. The viral video addressed “Ma” 20 times before its fiery crescendo:

Today, in this male‐dominant society, facing gender rules and mentalities, women have accepted defeat and set aside their weapons…. What, have you lost faith in your own power? Don't you have confidence in your own beloved daughter? Fie on women's power, fie on mothers committing female foeticide, and fie on their motherhood!

Materials condemned women seeking sex selection as “merciless,” “ferocious,” and “worse than monsters.” To escape demonization, feminine heroes had to overcome patriarchy through plucky, empowered action.

Like the threatened daughter, the backward woman or family crowded out structural factors. Despite Gujarat's obvious patriarchal inequities, male speakers instructed gatherings of women to performatively declare that “feminine power” was “unstoppable by anyone.” Messaging conveniently omitted market liberalization, rising costs, educational shifts, and limited social security as drivers of sex selection. Campaigns also elided how family planning governance had helped produce the backward “tradition” of prenatal sex selection, and how denunciations of the practice continually clashed with other biopolitical imperatives (cf. Kogacioglu, 2004). The fence around Mahesana District's health offices juxtaposed “Small Family” and “Save the Daughter” slogans, emblematizing the simultaneous promotion of general antinatalism and gender‐specific pronatalism. Governance messaging spliced out the double bind that made two “backward” practices—repeated childbearing and sex selection—the viable paths to the necessary son.

Despite such omissions, melodramas of backwardness aligned with existing moral sentimentality. Ideals of awareness and progress resonated, almost self‐evidently. Couples pursuing sex selection still valorized modern, ethical behavior. They often contrasted themselves against poorer, less educated, or more rural counterparts by disparaging high‐fertility son pursuit, or by insisting that *those* people perpetrated sex selection even more (statistics to the contrary notwithstanding).

But alignment between story and sociality was not necessarily sufficient to transform subjectivity or behavior. People did not fully embrace caricatures of themselves as villains. While acknowledging sex selection's badness, they situated it within pursuit of an overriding good: the necessary son (cf. Sandesara, 2024).

College professor Kinjal‐ben and business executive Gaurav‐bhai epitomized the partial participation that backwardness narratives induced. I met them when they opted for sex‐selective abortion after three daughters. While waiting for the procedure, Gaurav‐bhai sighed:

We ended up having to do this because we had no choice! We have three girls. And our family's educated—everyone, minimum college. We know all this shouldn't happen. There are advertisements ‐ on radio, TV, everything ‐ for Save the Daughter. There are things like that video of the little girl. But then, sometimes, there comes a time when you must become weak.

Gesturing to herself, Kinjal‐ben said:

Look, whatever else is true, a girl'll go away to her father‐in‐law's home. And then her father's name won't come after hers, her husband's will. And kids—the wife's name won't be the one after theirs, right? It's always the father's. His lineage keeps going.

Despite so much education, nobody changes this! A son remains with parents for life. A daughter doesn't…. So everyone prefers a boy. *You still need one son*.

Sex selection was not a problem of familial awareness and feeling. It was a problem of entanglement in the political and moral economies of patriarchy. Kinjal‐ben and Gauravi‐bhai were a college‐educated, media‐savvy couple. They had welcomed three daughters. They lamented their selective abortion even as it happened. But for them, as for countless others, an escapist fantasy was not enough to permit escape from inequitable structures.

## THE NARRATIVE GOVERNANCE OF LIFE

In anti‐sex selection melodrama, as in so much post‐2014, Gujarat became India's model. On January 22, 2015, Narendra Modi, now prime minister, proclaimed a national Save the Daughter campaign.

Modi's proclamation wove melodramatic tropes around threatened daughters, imbalanced populations, and backward families.^11^ He denounced “killing daughters in the womb” as sinful, an act of “citizens with eighteenth‐century mindsets,” a sign of “mental poverty” or “the disease of discrimination.” The world could not keep turning, Modi warned, if 1000 girls were not born for every 1000 boys. Quoting vernacular literature, he described daughters as “heaven on earth, the home of nations, and the honor of villages…” Dismissing “misconceptions” about economic support and out‐marriage, he insisted, “Once we have an attitude of equality toward sons and daughters in our minds, this sinful activity will end on its own.” He concluded by leading a prolonged oath to oppose sex selection, celebrate girls, and “spread the message of ‘Save the Daughter, Educate the Daughter’ throughout society.”

The speech epitomized the kind of biopolitical melodrama I found in Gujarati anti‐sex selection messaging. Sentimentalized narratives simplified a complex problem through good‐evil binaries, stock characters, emotional provocations, and redemptive plots. They evoked deep resonance, partly by rearticulating conservative moral elements (including the very gender‐kinship formations driving sex selection). They fostered shared feeling, bolstered institutional legitimacy, and marginalized radical critiques. But they did not necessarily transform dispositions or behaviors, for they failed to capture key aspects of lived sociality. Due to partial misalignments with everyday realities, an intervention thoroughly biopolitical in form, function, and content failed to foster its idealized version of life (cf. Stevenson, 2014, especially 3–4, 73, 86, 96; Mishtal, 2015, 246–320). Biopower was reduced to collective anxiety management, generating a felt sense of belonging, purpose, and action without changing practices.

The concept of biopolitical melodrama can generalize to governance of other potentially harmful practices. Facile victim‐villain‐hero narratives abound in interventions on gender issues like human trafficking (Vance, 2012) and violence against women (Kapur, 2002), or epidemics like cholera (Briggs & Mantini‐Briggs, 2002) and HIV (Fassin, 2012, 161–80). One could analyze responses to various other public health challenges, from drug use to environmental degradation, through the biopolitical melodrama lens. Whenever the governance of life targets behavior change—especially with “awareness” the goal and knowledge‐attitude‐behavior the operational paradigm—there exists the possibility for moralized, sentimentalized education centered on simplistic narratives. Because biopolitical melodrama inherently aligns with dominant ethical and emotional impulses, it can produce strong feelings of clarity and righteousness. But because biopolitical melodrama also inherently oversimplifies, projects reliant on it may fail to transform despite resonating. This is not, of course, a foregone conclusion; it is everywhere an ethnographic question.

In the Gujarati sex selection case, biopolitical melodrama's not‐quite‐paradoxes—encapsulation without accuracy, regulation without discipline, authority without efficacy, participation without change—suggest a nondeterministic approach to people's relationships with the governance of life. If the subjective limit of anti‐sex selection messaging is resonance rather than transformation, then examining the mechanisms of such messaging takes us beyond biopolitical determinism, into the moral and affective life of biopower. Most crucially, juxtaposing biopolitical narratives and pragmatic realities highlights how Gujarati public health messaging operates amid many other forces influencing reproductive behavior. Those forces include antinatalism, postcolonial meliorism, and commercialized biomedicine—biopolitical processes that spawned sex selection and that still clash with efforts against it. But Gujarati families also inhabit countless other regimes of life that influence reproduction: patrilivirilocal kinship, patriarchal respect, family‐based social security, market expansion, financial precarity, economic aspiration, casteism, classism, communalism, and more. These constitute the moral assemblage within which sex selection unfolds—the assemblage biopolitical melodrama (the phenomenon) obscures and biopolitical melodrama (the concept) highlights.

Given the broader contexts surrounding Gujarati sex selection, solutions likely require more radical transformation than biopolitical melodrama can catalyze. The state may be poorly positioned to drive change here. By contrast, caste associations, despite their inherent conservatism, may be able to take actions that significantly modify structural contexts. Many Gujarati caste associations already legislate norms and organize resource redistribution among members. Some have previously implemented reforms like joint weddings to reduce marriage costs. If associations instituted substantive programs to guarantee sonless parents financial security, respect, and robust connections with daughters, it might do more to change members’ dispositions and behaviors than moralistic messaging does.

Broadening from fantasies to pragmatic realities in this way helps us reframe behavior change problems as moral tragedies. Unlike melodramas (or romances), tragedies (or “weepies”) emphasize up‐and‐down, nonteleological engagement with everyday challenges (Anker, 2014, 227–56; Scott, 2004). The tragic mode captures the universal human condition of being trapped between incommensurable moral goods with no perfect choice (Mattingly, 2014, 145–58)—the scenario in sex selection and so many other public health challenges. This is not to say we should repackage melodramas into quasi‐mythical, cleanly delimited tragedies with heart‐rending outcomes; formal tragic stories have their own limitations (Brooks, 1995, 15, 205–7), and human sociality does not permit such neat encapsulation. Instead, the tragic mode provides a stance from which to approach everyday complexities and biopolitical interventions on them. Moreover, because the tragic mode can integrate the contingent social histories melodrama elides, it offers material for imagining an otherwise (Mattingly, 2014, 158–61). Every biopolitical melodrama calls for tragic reanalysis to uncover the competing truths it buries.

More broadly, if critique is the “art of not being governed like that and at that cost” (Foucault, 2007, 45), then critiquing the narrative governance of life is the art of not being narrated like that, and at that cost. Stories, as much as statistics or scatterplots, can structure the truths and interventions by which biopower attempts to order populations and bodies. Systematic analysis of biopower's narrative modes and devices—what we might call *biopolitical narration*—can illuminate how ethics, affect, and meaning suffuse the governance of life, and how such governance does or does not produce certain outcomes. Narrative analysis offers a fresh approach for understanding how people respond to biopower, and for imagining how stories might unfold differently.

## Supporting information



Supporting Information

## ENDNOTES

1 In everyday practice, Gujaratis engaging in and intervening against sex selection tended to assume an overdetermined, rigid sex/gender binary, and to mark little difference between “sex” and “gender.” In analyzing their sociomoral world, I take the binary as a pragmatic (albeit socially constructed) given and treat “sex” and “gender” essentially interchangeably. Elsewhere in my work, I break down these assumptions vis‐à‐vis sex selection.

2 Figures are from the 2011 census. (Due to the COVID‐19 pandemic, the 2021 census is still pending as of this writing.) Human biology seems to favor birth of fewer girls, leading to newborn sex ratios between 0.935 and 0.971 absent outside intervention (Waldron, 1998, 53–63). But a “female survival advantage,” in which girls better survive infancy and early childhood, can countervail the innate skew, balancing out child sex ratios (Waldron, 1998, 64–108). Historically, infanticide, neglect, and abuse have reversed the early‐life female survival advantage in India (Mitra, 1979, 17–24).

3 Following Gujarati conventions, I suffix kinship terms (‐ben [sister] and ‐bhai [brother]) to first names. All names are pseudonyms, except those of public figures. Translations from Gujarati and Hindi are mine.

4 Guilmoto (2015) provides a global overview. John and colleagues (2008, 52–87), Kaur and Kapoor (2021, 116–20), Purewal (2018), Unnithan‐Kumar (2011), and Visaria (2007, 154–60) discuss India.

5 But biopower can also collectivize (Gammeltoft, 2014, 59–76).

6 The history in this paragraph and the next two draws on Gupte (2003) and Ravindra (1993).

7 The judgment is at (2001) 5 SCC 577.

8 The Gujarati video is at <https://www.youtube.com/watch?v=KgdmsIEUEgE>. Accessed October 15, 2024.

9 The analysis of ventriloquy here builds on Basu (2010). See also Murphy (2017, 118–20).

10 Two families I spotlight had accepted multiple girls. But it is not as if melodrama resonated only with those predisposed to avoid sex selection; many pursuing sex selection after just one daughter exhibited similar responses.

11 The Hindi speech is at <https://www.narendramodi.in/text‐of‐pm‐shri‐narendra‐modis‐address‐at‐the‐ceremony‐to‐launch‐beti‐bachao‐beti‐padhao‐programme‐2815>. Accessed December 30, 2024.
