# Supplementary figures and images for: The narrative governance of life: Morality, melodrama, and the limits of biopower in western Indian efforts against sex selection
Source: Med Anthropol Q. 2024 Nov 29;39(2):e12901. doi: 10.1111/maq.12901 (PMC12140136; doi:10.1111/maq.12901)

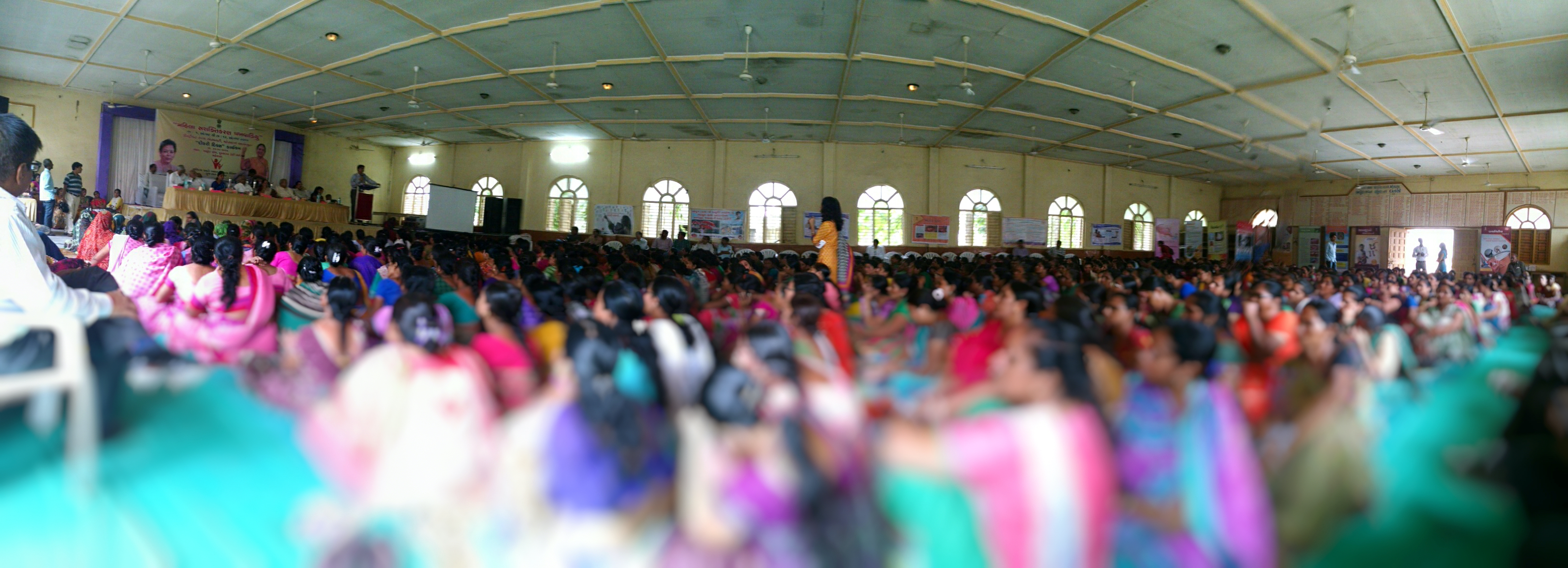

Supplement: Supplementary file 1 — Supporting Information [file MAQ-39-0-s001.jpg]
